# A genome-wide linkage analysis of alcoholism on microsatellite and single-nucleotide polymorphism data, using alcohol dependence phenotypes and electroencephalogram measures

**DOI:** 10.1186/1471-2156-6-S1-S17

**Published:** 2005-12-30

**Authors:** Chun Zhang, Simon Cawley, Guoying Liu, Manqiu Cao, Harley Gorrell, Giulia C Kennedy

**Affiliations:** 1 Genomics Collaborations Department, Affymetrix, Santa Clara, CA, 95051, USA; 2 Department of Algorithms and Data Analysis, Affymetrix, Santa Clara, CA, 95051, USA; 3 Bioinformatics Department, Affymetrix, Emeryville, CA, 94608, USA

## Abstract

The Collaborative Study on the Genetics of Alcoholism (COGA) is a large-scale family study designed to identify genes that affect the risk for alcoholism and alcohol-related phenotypes. We performed genome-wide linkage analyses on the COGA data made available to participants in the Genetic Analysis Workshop 14 (GAW 14). The dataset comprised 1,350 participants from 143 families. The samples were analyzed on three technologies: microsatellites spaced at 10 cM, Affymetrix GeneChip^® ^Human Mapping 10 K Array (HMA10K) and Illumina SNP-based Linkage III Panel. We used ALDX1 and ALDX2, the COGA definitions of alcohol dependence, as well as electrophysiological measures TTTH1 and ECB21 to detect alcoholism susceptibility loci. Many chromosomal regions were found to be significant for each of the phenotypes at a *p*-value of 0.05. The most significant region for ALDX1 is on chromosome 7, with a maximum LOD score of 2.25 for Affymetrix SNPs, 1.97 for Illumina SNPs, and 1.72 for microsatellites. The same regions on chromosome 7 (96–106 cM) and 10 (149–176 cM) were found to be significant for both ALDX1 and ALDX2. A region on chromosome 7 (112–153 cM) and a region on chromosome 6 (169–185 cM) were identified as the most significant regions for TTTH1 and ECB21, respectively. We also performed linkage analysis on denser maps of markers by combining the SNPs datasets from Affymetrix and Illumina. Adding the microsatellite data to the combined SNP dataset improved the results only marginally. The results indicated that SNPs outperform microsatellites with the densest marker sets performing the best.

## Background

Alcoholism is a complex disorder in which multiple genes may contribute to the risk [1]. To address this complexity, the Collaborative Study on the Genetics of Alcoholism (COGA) researchers designed a large-scale family study and collected multiple alcoholism and alcohol-related phenotypes from the participants.

ALDX1, the primary COGA definition of alcohol dependence, requires a person to meet both DSM-III-R criteria and the Feighner criteria [1,2]. Linkage analyses using ALDX1 have provided evidence of linkage on chromosome 1, 2, and 7 [3,4]. Highly heritable electrophysiological variables, such as electroencephalography (EEG) and event-related potentials (ERPs), have been used to identify the genes that affect brain activities related to alcoholism. Data from the Eyes Closed Resting EEG experiment (ECB21) have revealed a strong linkage on chromosome 4 [5,6]. In further studies, the far frontal left side channel measure extracted from the target case of the Visual Oddball experiment for 4 electrode placements (TTTH1) revealed a strong linkage on chromosome 7 [7]. In these studies a 10-cM map of microsatellites was used for the initial scan. The recent addition of singe nucleotide polymorphisms (SNPs) within these linkage regions has improved the resolution of the mapping results [1,7].

The microsatellite-based screening approach has been used successfully for mapping Mendelian diseases. However, this technique has been proven to be unreliable for complex genetic diseases [8,9]. It has been suggested that a 1–2 cM map of moderately polymorphic biallelic markers would be more powerful than a 5–10 cM map of microsatellite screening sets [10]. If that is the case, the recently developed, high-density oligonucleotide array-based, whole-genome sampling analysis approach [11] should provide an ideal set of genotype data for a whole genome scan.

The COGA dataset provided to participants at GAW14 included data from 1,350 participants from 143 families. The genotype dataset included data produced by a 10-cM map of microsatellites, Affymetrix GeneChip^® ^Human Mapping 10 K Array (HMA10K), and Illumina SNP-based Linkage III Panel. To identify susceptibility regions for alcoholism, we performed a genome-wide multipoint linkage analysis using alcohol dependence phenotypes ALDX1, ALDX2 (diagnosed by DSM-IV criteria), and quantitative traits TTTH1 and ECB21. The performance of microsatellites, Affymetrix HMA10K Array, and Illumina Linkage III Panel were compared in terms of information content, identified linkage regions and the 1-LOD support interval of the regions.

## Methods

### Map construction

The 10-cM microsatellite maps contained 328 microsatellites of which 309 have unique locations on the deCode high-resolution genetic maps. To map the SNPs, we first obtained the physical locations from build 34 of the human genome dbSNP database at the National Center for Biotechnology Information (NCBI). We then interpolated the genetic map locations using the microsatellite with unique physical locations in deCode genetic maps. 11,050 Affymetrix SNPs and 4,700 Illumina SNPs with unique sex-averaged genetic map locations were used in our study. We also created an even denser map of markers by combining Affymetrix and Illumina SNPs (Comb2). In addition, we combined Affymetrix and Illumina SNPs with the microsatellite data (Comb3) to determine the contribution of microsatellite markers.

### Genotype error detection

The datasets were prepared with PEDCHECK [12] to remove Mendelian inconsistencies. A small number of erroneous genotypes can reduce the power of linkage analysis [13-15]. We therefore used MERLIN [16] to eliminate the genotypes with unlikely recombination patterns (0.39% for microsatellites, 0.16% for Affymetrix SNPs, 0.13% for Illumina SNPs).

### Linkage analysis

Information content (IC) measures how much of the inheritance information can be extracted from available genotype data. It closely predicts the power of a map to detect linkage [10]. We used MERLIN to calculate IC at every marker locus.

Alcohol dependence phenotypes ALDX1 and ALDX2 include five categories: no information; pure unaffected; never drank; unaffected with some symptoms; affected. We treated "never drank" as "no information", and combined "pure unaffected" and "unaffected with some symptoms" as "unaffected." We performed nonparametric linkage (NPL) analysis based on the identity-by-descent (IBD) sharing among affected individuals in a pedigree. We used MERLIN to calculate NPL_all _[17] and the corresponding nonparametric LOD scores based on the linear model [18]. NPL scores are generally regarded as conservative [17,18]. The nonparametric LOD scores give more accurate *p*-values and can be used to construct 1-LOD support intervals [9]. For this reason we used the nonparametric LOD scores in our analysis. We chose significance level 0.05 to report linkage regions.

We conducted variance components analyses on the log transformed quantitative traits TTTH1 and ECB21, adjusting for age and sex. Heritability of the traits and the LOD scores at every marker locus were calculated by using MERLIN.

### Computational implementation

MERLIN is a software package designed for dense genetic maps in pedigree data. It efficiently implements the Lander-Green algorithm [19] by using sparse binary trees to represent gene flow. We modified the tree structure in the source code of MERLIN and adjusted different compilation options to improve efficiency on highly dense maps and extensive pedigrees by 25% on a 32-bit UNIX machine and 50% on a 64-bit UNIX machine.

## Results

### Summary of the maps

The summary statistics for the data are shown in Table 1. Among the three data sets, Affymetrix SNPs have the highest and the most uniform density across the genome. Due to the sparseness of the microsatellites, there is only slight difference in density between the combined SNPs and the combined SNPs and microsatellites.

### Information content

Microsatellites had the lowest mean and highest standard deviation (SD) of genome-wide IC due to the limited coverage of the genome (Table 2). With the densest map, Affymetrix SNPs produced the highest mean and lowest SD as well as a lowest inter-quartile range and most narrow range of IC. This indicates a uniform and robust distribution of IC across the genome. The same trend remained for the combined map with microsatellites and SNPs (Comb3). The combined SNPs data (Comb2) show similar IC compared with Comb3 (Table 3). These results show that density plays a key role in extracting inheritance information from the available genotype data.

### Linkage analysis with alcohol dependence phenotypes ALDX1 and ALDX2

Both ALDX1 and ALDX2 phenotypes showed significant linkage on chromosome 7 (96–106 cM) and chromosome 10 (149–176 cM) in all the datasets. In the SNP datasets, both phenotypes detected the same region on chromosome X (30–46 cM), although the LOD scores for ALDX2 were much less significant (Table 4, Table 5).

**Figure 1:**
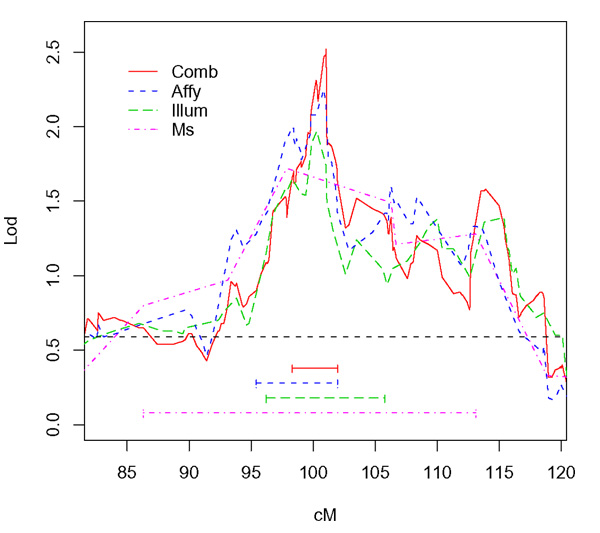
**Linkage region on chromosome 7**. LOD scores of the linkage region (83–119 cM) for microsatellite markers (pink), Affymetrix SNPs (blue), Illumina SNPs (green) and the combined data (red). Horizontal dashed line is the critical value at point-wise significance level of 0.05. Bars on the bottom indicate 1-LOD intervals for this region in microsatellites (pink), Affymetrix SNPs (blue), Illumina SNPs (green), and the combined data (red).

Our analyses also detected linkage regions unique to each phenotype. For ALDX1, linkage was detected on chromosome 2, 7, 10, and 11 (Table 4) in all of the data sets. Additional linkage regions on chromosome 1, 6, 9, 12, 13, 18, and X were detected in the SNP datasets. Consistent with previous literature [3,4], the most significant linkage region in the combined data (Comb3) was located on chromosome 7 (Figure 1). In the Affymetrix panel, the highest maximum LOD score was 2.25 at 100.871 cM (Table 4). The 1-LOD intervals for this linkage region (Affymetrix: 6.551 cM; Illumina: 9.557 cM; microsatellites: 26.80 cM) revealed significant difference between microsatellites and SNPs. An even higher maximum LOD score of 2.52 at 101.049 cM with a narrower 1-LOD interval 3.647 (Figure 1) was achieved with a denser map in the combined datasets (Comb3). On chromosome 1 and 2 we found linkage regions adjacent to the significant regions reported in [3,4] in both SNP datasets. In contrast, we did not find any evidence of linkage to these two regions in the microsatellite data. Analyses using ALDX1 on selected chromosomes of the Comb2 and Comb3 data showed almost identical results.

For ALDX2, linkage regions on chromosome 1 (247–259 cM), 7 (96–106 cM), 10 (141–176 cM), and 17 (30–53 cM) were significant at a level of 0.05 in all the three datasets. Other significant regions on chromosome 2, 3, 6, 7, 9, 10, and X were detected in SNP data sets but were not present in the microsatellite data (Table 5).

### Linkage analysis with EEG measures TTTH1 and ECB21

The kurtosis values of the log transformed TTTH1 and ECB21 are -0.13 and -0.44, respectively, indicating no significant deviation from the normal distribution. Therefore, these trait values were acceptable for use in variance components analysis.

The estimate of heritability, after adjusting for age and sex, is 35.17% for TTTH1. The most significant region on chromosome 7 (112–153 cM: LOD score 1.47 for Affymetrix SNPs, 2.01 for Illumina SNPs, and 2.44 for microsatellites) overlaps with the one reported by Jones et al. [7], but the heritability is lower. The difference in heritability values could be due to different sample structure or different algorithms (Jones et al. [7] performed the variance components analyses using the t-distribution option of SOLAR) used in these two studies.

The estimate of heritability for ECB21 is 55.54% after adjusting for age and sex. One of the linkage regions on chromosome 4 (58–79 cM: LOD score 1.10 for Affymetrix, 1.25 for Illumina, and 1.40 for microsatellites) overlaps with the highly significant linkage region found in previous studies [5,6]. The most significant region is on chromosome 6 (169–185 cM: LOD score 2.18 for Affymetrix, 2.11 for Illumina, and 0.71 for microsatellites).

## Discussion

Based on the COGA data provided to participants at GAW14, we have presented a NPL analysis for alcohol dependence phenotypes ALDX1 and ALDX2, and a variance component analysis for EEG measures TTTH1 and ECB21. Our results confirmed some of the linkage findings in previous studies [3-7]. The increased density of the SNP data extends the number of regions detected and increases the resolution of the linkage results.

In our study, we used a *p*-value of 0.05 as the significance level to report linkage regions. However, in order to define true linkage and explain the inconsistencies among the results of different datasets, it is important to choose a level of genome-wide significance. Commonly used resampling-based and gene-drop simulation approaches are computationally intensive and do not lend themselves to the analysis of the large amount of data in this study. We are investigating a more efficient Monte Carlo procedure to assess genome-wide significance in linkage analysis [20].

Our results show that a denser map can be more powerful for linkage analysis. IBD sharing based linkage analysis algorithms usually assume linkage equilibrium between the markers and the strong linkage disequilibrium between closely adjacent markers could potentially introduce false linkage results [21,22]. A study using Affymetrix HMA10K arrays indicated that there was no substantial difference in the results when SNPs in linkage disequilibrium are either retained or removed [15].

This study represents an extensive performance comparison of three different platforms (microsatellite markers, Affymetrix HMA10K Array, and Illumina Linkage III Panel) in a series of linkage analyses for alcoholism. The high density and the robust performance of SNPs make the whole-genome scan a desirable approach for linkage analysis. This new approach may bring a renewed power to IBD sharing based linkage analysis.

## Abbreviations

COGA: Collaborative Study on the Genetics of Alcoholism

ERP: Event-related potential

GAW: Genetic Analysis Workshop

IBD: Identity by descent

IC: Information content

NPL: Nonparametric linkage

SNP: Single-nucleotide polymorphism

## Authors' contributions

CZ carried out the statistical analysis and drafted the manuscript. SC assembled the Affymetrix linkage data, conducted qualify control and provided useful comments for the study. GL constructed unique genetic maps for all the data sets. MC generated data from the Affymetrix HMA10K array. HG modified MERLIN program to reduce the memory usage. GCK conceived of the study and participated in its design and coordination.

## Figures and Tables

**Table 1:** Summary of the maps

Map	Number of markers	Mean spacing (SD) (in cM)	Mean heterozygosity (range)
Microsatellite	309	10.820 (8.650)	0.715 (0.134–0.922)
Affymetrix	11,049	0.328 (0.619)	0.340 (0–0.720)
Illumina	4,700	0.786 (1.182)	0.440 (0.100–0.550)
Comb3^a^	16,058	0.230 (0.405)	N/A
Comb2^b^	15,749	0.234 (0.414)	N/A

^a^Comb2: combined Affymetrix and Illumina SNP data.

^b^Comb3: combined microsatellite markers, and Affymetrix and Illumina SNP data.

**Table 2:** Summary of IC

	Minimum	Maximum	Mean	SD	Interquartile range
Microsatellite	0.3394	0.9569	0.7937	0.0990	0.1306
Affymetrix	0.5239	0.9678	0.9421	0.0258	0.0166
Illumina	0.3765	0.9606	0.9189	0.0289	0.0252
Comb3^a^	0.7592	0.9908	0.9707	0.015	0.0131

^a^Comb3: combined microsatellite marker and both Affymetrix and Illumina SNP data.

**Table 3:** Comparison of IC and density for combined data

	Chr 2		Chr 6		Chr 7		Chr X	
				
	Mean IC (SD)	Spacing (SD)	Mean IC (SD)	Spacing (SD)	Mean IC (SD)	Spacing (SD)	Mean IC (SD)	Spacing (SD)
Comb3^a^	0.975 (0.01)	0.191 (0.30)	0.974 (0.01)	0.181 (0.33)	0.976 (0.01)	0.217 (0.36)	0.947 (0.03)	0.396 (0.72)
Comb2^b^	0.958 (0.01)	0.196 (0.31)	0.957 (0.01)	0.184 (0.33)	0.956 (0.01)	0.225 (0.37)	0.931 (0.03)	0.407 (0.74)

^a^Comb3: combined data set of microsatellites, Affymetrix and Illumina SNPs.

^b^Comb2: combined data of Affymetrix and Illumina SNPs.

**Table 4:** Summary of linkage results for ALDX1

		Affymetrix SNPs		Illumina SNPs		Microsatellites		Comb3	
					
Chr	Location (cM)	LOD (*p*)	IC/Chr	LOD (*p*)	IC/Chr	LOD (*p*)	IC/Chr	LOD (*p*)	IC/Chr
1	(138–148)	1.060 (0.014)	0.942 (0.02)	1.330 (0.007)	0.932 (0.02)		0.832 (0.07)	0.950 (0.020)	0.972 (0.01)
2	(10–30)	1.140 (0.011)	0.948(0.02)	1.740 (0.002)	0.934 (0.02)	1.51 (0.004)	0.845 (0.08)	1.440 (0.005)	0.975 (0.01)
	(114–125)	1.100 (0.012)		0.700 (0.040)				1.080 (0.013)	
	(134–147)	1.100 (0.012)		1.540 (0.004)				1.180 (0.010)	
	(228–252)	1.720 (0.002)						1.660 (0.003)	
6	(20–24)	0.810 (0.030)	0.947 (0.02)	0.660 (0.040)	0.935 (0.02)		0.803 (0.09)	0.940 (0.020)	0.974 (0.01)
	(183–190)			2.150 (0.0008)				1.910 (0.002)	
7	(3–19)	1.350 (0.006)	0.944 (0.03)	2.3200 (0.0005)	0.933 (0.02)	0.69 (0.04)	0.898 (0.05)	1.720 (0.002)	0.976 (0.01)
	(29–37)	1.170 (0.010)		0.760 (0.030)				1.060 (0.014)	
	(54–62)	0.980 (0.020)		0.740 (0.030)		1.22 (0.009)		1.230 (0.009)	
	**(83–119)**	**2.2500 (0.0006)**		**1.97 (0.0013)**		**1.72 (0.002)**		**2.5200 (0.0003)**	
9	(141–142)	0.660 (0.040)	0.947 (0.02)	0.690 (0.040)	0.926 (0.02)		0.782 (0.10)	0.770 (0.030)	0.974 (0.01)
10	(122–128)	0.750 (0.030)	0.943 (0.02)	0.650 (0.040)	0.924 (0.03)		0.664 (0.08)		0.967 (0.01)
	(149–179)	1.9900 (0.0012)		1.750 (0.002)		0.94 (0.02)		1.770 (0.002)	
11	(117–130)	1.280 (0.008)	0.946 (0.02)	1.030 (0.015)	0.924 (0.02)	1.39 (0.006)	0.709 (0.07)	1.450 (0.005)	0.968(0.01)
12	(0–5)	1.150 (0.011)	0.944 (0.02)		0.929 (0.03)		0.816 (0.08)		0.974 (0.01)
	(121–125)	0.830 (0.030)		0.78 (0.03)				0.800 (0.030)	
	(167–172)	0.700 (0.040)		0.930 (0.020)					
13	(81–102)	1.550 (0.004)	0.945 (0.02)	1.220 (0.009)	0.931 (0.02)		0.802 (0.10)	1.460 (0.005)	0.972 (0.01)
17	(34–36)		0.922 (0.05)		0.914 (0.03)	1.110 (0.012)	0.694 (0.08)	0.750 (0.030)	0.961 (0.02)
	(121–123)			0.800 (0.030)					
18	(93–107)	0.960 (0.020)	0.940 (0.02)	0.720 (0.030)	0.918 (0.04)		0.548 (0.06)	0.910 (0.020)	0.959 (0.02)
X	(30–46)	2.0500 (0.0011)	0.915 (0.03)	1.520 (0.004)	0.902 (0.08)		0.744 (0.17)	1.460 (0.005)	0.947 (0.03)

Summary of maximum LOD scores and the corresponding *p*-values (<0.05) for linkage regions for alcohol dependence phenotype ALDX1. Results are shown for microsatellites, Affymetrix SNPs, Illumina SNPs and the combined data (Comb3). The mean and standard deviation of IC are given for each chromosome. Bold text indicate the most significant region on chromosome 7.

**Table 5:** Summary of linkage results for ALDX2

		Affymetrix SNPs		Illumina SNPs		Microsatellite	
				
Chr	Location (cM)	LOD (*p*)	IC/Chr	LOD (*p*)	IC/Chr	LOD (*p*)	IC/Chr
1	42–44		0.942 (0.02)	0.73 (0.03)	0.932 (0.02)	0.68 (0.04)	0.832 (0.07)
	247–259	0.81 (0.03)		0.72 (0.03)		0.68 (0.04)	
2	19–23		0.948 (0.02)	0.86 (0.02)	0.934 (0.02)	0.75 (0.03)	0.845 (0.08)
	226–247	1.49 (0.004)		1.53 (0.004)			
3	90–93	0.99 (0.02)	0.947 (0.01)	0.70 (0.04)	0.938 (0.02)		0.755 (0.08)
							
6	171–189	1.49 (0.004)	0.947 (0.02)	1.69 (0.003)	0.935 (0.02)		0.803 (0.09)
7	3–9	1.01 (0.02)	0.944 (0.03)	2.25 (0.0006)	0.933 (0.02)		0.898 (0.05)
	59–60	0.64 (0.04)				0.82 (0.03)	
	96–106	1.22 (0.009)		0.85 (0.02)		0.83 (0.03)	
	108–116	0.99 (0.02)		0.87 (0.02)			
9	133–155	1.28 (0.008)	0.947 (0.02)	1.50 (0.004)	0.926 (0.02)		0.782 (0.10)
10	47–49	0.75 (0.03)	0.943 (0.02)		0.924 (0.03)		0.664 (0.08)
	120–130	1.18 (0.01)		1.33 (0.007)			
	141–176	1.97 (0.0013)		1.78 (0.002)		0.74 (0.03)	
12	167–172		0.944 (0.02)	0.94 (0.02)	0.929 (0.03)		0.816 (0.08)
17	30–53	1.44 (0.005)	0.922 (0.05)	1.39 (0.006)	0.914 (0.03)	1.83 (0.002)	0.694 (0.08)
X	30–46	1.07 (0.013)	0.915 (0.03)	0.89 (0.02)	0.902 (0.08)		0.744 (0.17)

Summary of maximum LOD scores and the corresponding p-values (<0.05) for linkage regions for alcohol dependence phenotype ALDX2. Results are shown for microsatellites, Affymetrix SNPs and Illumina SNPs. The mean and standard deviation of IC are given for each chromosome.
